# Soybean antiviral immunity conferred by dsRNase targets the viral replication complex

**DOI:** 10.1038/s41467-019-12052-5

**Published:** 2019-09-27

**Authors:** Kazuhiro Ishibashi, Masayasu Saruta, Takehiko Shimizu, Miao Shu, Toyoaki Anai, Kunihiko Komatsu, Naohiro Yamada, Yuichi Katayose, Masayuki Ishikawa, Masao Ishimoto, Akito Kaga

**Affiliations:** 1 Plant and Microbial Research Unit, Division of Plant and Microbial Sciences, Institute of Agrobiological Sciences, National Agriculture and Food Research Organization, 2-1-2 Kannondai, Tsukuba, Ibaraki 305-8602 Japan; 2 Crop Breeding and Food Functional Components Division, Western Region Agricultural Research Center, National Agriculture and Food Research Organization, 1-3-1 Senyu-cho, Zentsuji-shi, Kagawa 765-8508 Japan; 3 Soybean and Field Crop Applied Genomics Research Unit, Institute of Crop Science, National Agriculture and Food Research Organization, 2-1-2 Kannondai, Tsukuba, Ibaraki 305-8518 Japan; 4 Faculty of Agriculture, Saga University, 1 Honjo-machi, Saga, 840-8502 Japan; 5 Research Team for Crop Cold Tolerance, Hokkaido Agricultural Research Center, National Agriculture and Food Research Organization, Hitsujigaoka 1, Toyohira, Sapporo, Hokkaido 062-8555 Japan; 6 Nagano Vegetable and Ornamental Crops Experiment Station, 1066-1, Soga, Shiojiri, Nagano 399-6461 Japan; 7 Advanced Genomics Breeding Section, Institute of Crop Science, National Agriculture and Food Research Organization, 1-2 Ohwashi, Tsukuba, Ibaraki 305-8634 Japan; 8 Present Address: Soybean Breeding Unit, Institute of Crop Science, National Agriculture and Food Research Organization, 2-1-2 Kannondai, Tsukuba, Ibaraki 305-8518 Japan; 9 Present Address: Advanced Genomics Breeding Section, Institute of Crop Science, National Agriculture and Food Research Organization, 2-1-2 Kannondai, Tsukuba, Ibaraki 305-8518 Japan; 10 Present Address: Crop Breeding and Food Functional Components Division, Western Region Agricultural Research Center, National Agriculture and Food Research Organization, 1-3-1 Senyu-cho, Zentsuji-shi, Kagawa 765-8508 Japan; 11 Present Address: Department of Planning and Coordination, National Agriculture and Food Research Organization, 3-1-1 Kannondai, Tsukuba, Ibaraki 305-8517 Japan; 12 Present Address: Division of Basic Research, Institute of Crop Science, National Agriculture and Food Research Organization, 3-1-1 Kannondai, Tsukuba, Ibaraki 305-8517 Japan

**Keywords:** Molecular engineering in plants, Agricultural genetics, Biotic

## Abstract

Eukaryotic positive-strand RNA viruses replicate their genomes in membranous compartments formed in a host cell, which sequesters the dsRNA replication intermediate from antiviral immune surveillance. Here, we find that soybean has developed a way to overcome this sequestration. We report the positional cloning of the broad-spectrum soybean mosaic virus resistance gene *Rsv4*, which encodes an RNase H family protein with dsRNA-degrading activity. An active-site mutant of Rsv4 is incapable of inhibiting virus multiplication and is associated with an active viral RNA polymerase complex in infected cells. These results suggest that Rsv4 enters the viral replication compartment and degrades viral dsRNA. Inspired by this model, we design three plant-gene-derived dsRNases that can inhibit the multiplication of the respective target viruses. These findings suggest a method for developing crops resistant to any target positive-strand RNA virus by fusion of endogenous host genes.

## Abstract (web summary)

*Soybean mosaic virus* (SMV) is a potyvirus that reduces soybean yield and seed quality worldwide. Here, the authors reveal that the resistance gene *Rsv4* encodes an RNase H family protein with dsRNA-degrading activity, and it can enter the viral replication compartment and degrade viral dsRNA.

## Introduction

Eukaryotes have evolved several layers of antiviral defense systems. Double-stranded (ds) RNA is a virus-specific molecular pattern recognized by hosts that triggers innate immunity or RNA silencing^1^. In plants, dsRNAs produced by either viral or plant endogenous RNA-dependent RNA polymerases (RdRp) are processed by the Dicer-like RNase III proteins to generate 21-nt to 24-nt small interfering (si) RNAs. siRNAs are incorporated into Argonaute proteins to make an RNA-induced silencing complex (RISC), which cleaves RNAs having sequences complementary to the siRNA^2^. To counteract RNA silencing by the host, viruses encode suppressors of RNA silencing, many of which bind siRNA and prevent RISC formation^2^.

In addition to the RNA-silencing-mediated basal resistance, plants have genes that confer resistance to specific viruses. Most virus resistance genes in plants encode nucleotide binding site–leucine-rich repeat (NB-LRR) proteins that elicit a defense reaction upon recognition of pathogens^3^. Other virus resistance genes encode structurally unrelated proteins with various functions^3^. Resistance genes have been introduced into crops to protect them from viral diseases; however, the available resistance genes cover only a limited range of viruses.

The genus *Potyvirus* in the family *Potyviridae*, a family of positive-strand RNA viruses, includes many economically important crop viruses such as *Potato virus Y* (PVY), *Turnip mosaic virus* (TuMV), and *Plum pox virus* (PPV). Potyviruses are transmitted by aphids, and their genome encodes a large polyprotein that is processed into ten or eleven proteins^4^. To protect from potyviruses, resistance genes have been introduced into many crop cultivars such as potato^5^, pepper^6^, papaya^7^, plum^8^, *Brassica* crops^9^, and legumes^10^.

*Soybean mosaic virus* (SMV) is a potyvirus that reduces soybean yields and seed quality worldwide^11,12^. Four dominant SMV resistance genes have been found in soybean accessions and introduced into commercial cultivars. *Rsv1*, *Rsv3*, and *Rsv5* are strain-specific and are suggested to encode NB-LRR proteins^13–17^. In contrast, *Rsv4* confers broad-spectrum resistance to SMV strains through an atypical mechanism that delays SMV multiplication^18^. In this study, we aim to clone the *Rsv4* gene and elucidate the resistance mechanism. The mechanism of *Rsv4*-mediated SMV resistance deepens our knowledge of the virus–host arms race and inspire us to develop a method to engineer virus-resistant organisms by fusion of endogenous host genes.

## Results

### Positional cloning of the SMV resistance gene *Rsv4*

**Fig. 1:**
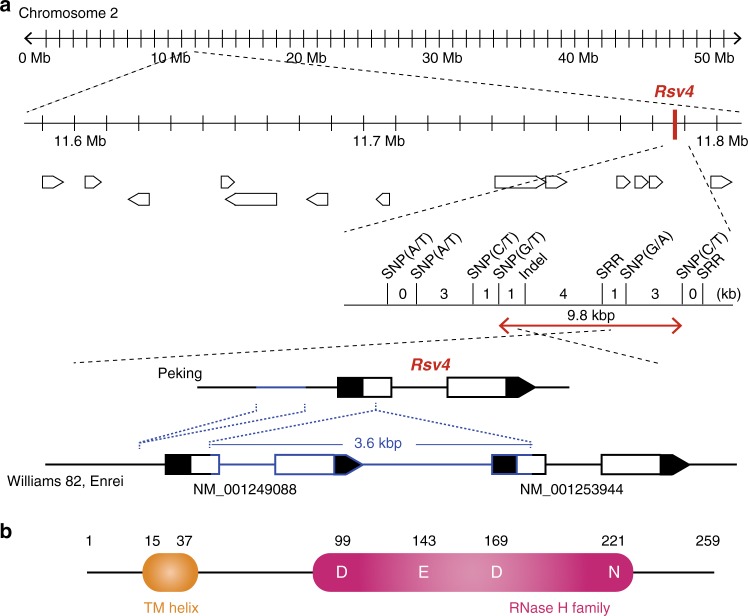
Positional cloning of *Rsv4*. **a** Fine mapping of *Rsv4*. The gene structures in the Peking, Williams 82, and Enrei genomes are shown at the bottom. Indels are indicated in blue. Black boxes are untranslated regions. **b** Predicted Rsv4 protein structure. Amino acid positions of the transmembrane (TM) helix and active-site residues of the RNase H family domain are shown

**Fig. 2:**
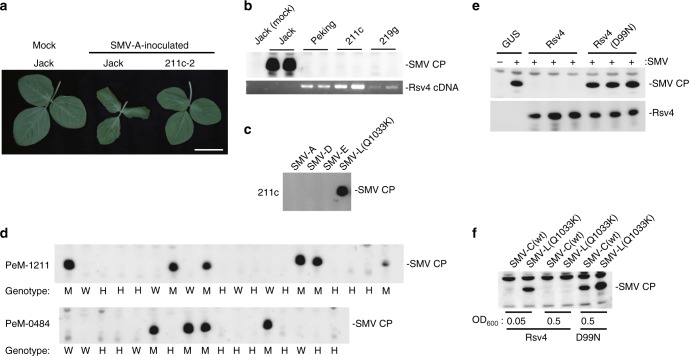
*Rsv4* encodes an RNase H family protein. **a** Symptoms of SMV-inoculated soybean plants. 211c-2 is a transgenic line derived from susceptible cv. Jack, overexpressing *Rsv4* from Peking. Bar = 5 cm. **b** SMV coat protein (CP) accumulation in control and transgenic soybean plants. 211c expresses *Rsv4* cDNA driven by the cauliflower mosaic virus 35 S promoter, while 219 g carries a genomic DNA fragment of Peking containing the putative native promoter of *Rsv4*. SMV CP accumulation in non-inoculated upper leaves was detected by western blotting at 10 days post-inoculation (dpi) (upper panel). *Rsv4* mRNA was detected by RT-PCR (lower panel). Lanes represent individual plants. **c** SMV strains SMV-A, -D, -E, and -L(Q1033K) were used to inoculate 211c, and CP accumulation was analyzed as in panel **b**. **d** Co-segregation of PeM-1211 and PeM-0484 (identical nonsense mutations in Peking *Rsv4* gene) and SMV-susceptible phenotype. W wild-type, M mutant-type, H heterozygous. **e** Rsv4, but not the Rsv4(D99N) mutant protein, inhibits SMV multiplication in *N. benthamiana* leaves. SMV CP accumulation was detected by western blotting at 5 dpi. **f** SMV-L(Q1033K) is less sensitive to Rsv4 than SMV-C. *Agrobacterium* strains containing constructs to express Rsv4 or Rsv4(D99N) were infiltrated at the indicated concentrations. 1 day after infiltration, *Agrobacterium* harboring SMV cDNA was infiltrated at OD_600_ = 0.005. CP accumulation was detected at 7 dpi. The source data of Fig. 2b-f are provided as a Source Data file

Here, we report positional cloning of *Rsv4* (Fig. 1a, Supplementary Fig. 1). Using 9320 progenies of a cross between soybean cultivars Peking (*Rsv4*; resistant) and Enrei (*rsv4*; susceptible), we mapped the gene within a 9.8-kbp region on soybean chromosome 2, adjacent to the *Rsv4* region previously mapped by other groups^19–21^. In this region of Enrei and the SMV-susceptible cultivar Williams 82, two tandemly repeated homologous open reading frames (ORFs; NM_001249088 and NM_001253944) were found (Fig. 1a). Both ORFs contain an intron and encode RNase H-like proteins (Supplementary Fig. 1c, d). In contrast, a 3.6-kbp deletion was found in this region of the Peking genome, and only a single ORF that encodes a 259-amino-acid protein was found (Fig. 1a, Supplementary Fig. 1). The predicted protein from the Peking ORF has an RNase H–like domain and a transmembrane helix (Fig. 1b). Transgenic soybean plants overexpressing this RNase H family protein from Peking showed resistance to all tested SMV strains except for SMV-L(Q1033K), a mutant SMV that has an amino acid substitution in the P3 cistron and can multiply more rapidly than its parental strain in *Rsv4* plants^22^ (Fig. 2a–c). Thus, this RNase H family protein inhibits SMV multiplication with the same virus specificity seen in *Rsv4* soybean lines. Following ethyl methanesulfonate (EMS) mutagenesis of Peking, we isolated four independent mutant alleles of this RNase H family gene and found that three of them conferred recessive susceptibility to strain SMV-C (Fig. 2d, Supplementary Table 1, Supplementary Fig. 2). We conclude that the *Rsv4* gene encodes this RNase H family protein.

**Fig. 3:**
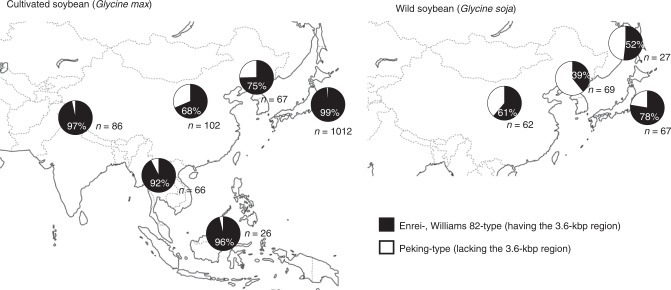
Geographical distribution of genetic diversity of the *Rsv4* locus. The presence or absence of the 3.6-kbp region in *Rsv4* in cultivated soybean (*Glycine max*) and wild soybean (*G. soja*) are shown in black or white, respectively. The number of investigated plants and percentage of plants having the 3.6-kbp region are indicated for each geographical area. See also Supplementary Data 1

The 3.6-kbp deletion identified in resistant cultivar Peking was rare in cultivated soybean but was found in Chinese and Korean soybean (*Glycine max*) landraces and more frequently in *Glycine soja*, the wild progenitor of soybean (Fig. 3). All of the resistant accessions we examined contained the 3.6-kbp deletion. On the other hand, there were susceptible accessions both with and without the 3.6-kbp deletion, indicating that it did not always confer SMV resistance (Supplementary Fig. 3). We further classified 63 soybean accessions that carried the 3.6-kbp deletion into 16 types based on amino acid sequence (Supplementary Figs. 3 and 4). A close relationship was observed between amino acid sequence and a degree of SMV CP accumulations, providing supporting evidence that the gene encoding the RNase family protein is *Rsv4*. The predicted Rsv4 protein sequences of Beeson (PI548510)^23^ and PI88788^18^, which are both resistant to SMV but reported to have different *Rsv4* alleles, differed at 3 and 16 positions, respectively, from that of Peking and V94-5152 (PI596752)^19^ (Supplementary Fig. 4). The SMV-C resistance of Fukusennari was mapped to the *Rsv4* region by using progenies of a cross with Enrei, but its sequence is different from those of Peking, Beeson, and PI88788 (Supplementary Figs. 3 and 4). The syntenic regions in other legume genomes contain complex arrangements of RNase H genes, suggesting the prevalence of genomic rearrangements at this locus (Supplementary Fig. 5). While RNase H proteins are universally encoded in plant genomes, the N-terminal transmembrane helix, such as found in Rsv4, is unique to legume RNase H proteins.

### Rsv4 is a dsRNase interacting with SMV replication machinery

The Rsv4 protein contains a DEDN motif in which the fourth residue of the DEDD motif in the active site of many RNase H proteins^24^ is changed from aspartate to asparagine (Fig. 1b). We introduced a mutation into Rsv4 to substitute the first D of the DEDN motif with N (D99N). Transient expression of Rsv4 by agroinfiltration inhibited SMV multiplication in *Nicotiana benthamiana* but the Rsv4(D99N) mutant did not (Fig. 2e), suggesting that resistance depends on an activity of the RNase H–like domain. When *Agrobacterium* containing a wild-type *Rsv4* construct was infiltrated at high concentrations, virus multiplication was inhibited even for SMV-L(Q1033K) (Fig. 2f), indicating that overexpression of Rsv4 can increase the durability of the resistance. In the *N. benthamiana* transient overexpression system, one of the two ORFs from Jack (susceptible), NM_001249088, inhibited SMV multiplication while the inhibitory activity was weaker than Rsv4 when *Agrobacterium* concentration was low (Supplementary Fig. 6a). NM_001249088 mRNA was expressed at similar or slightly higher levels to *Rsv4* in soybean leaves (Supplementary Fig. 6b). These results suggest that NM_001249088 encodes a functional antiviral protein but that the effects of its endogenous expression in soybean have been overcome by SMV.

**Fig. 4:**
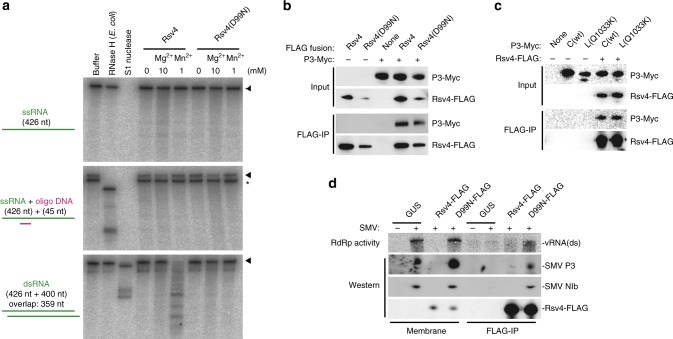
Rsv4 is a Mn^2+^-dependent dsRNase that associates with SMV replication machinery. **a** Activity of Rsv4. Purified Rsv4 protein or Rsv4 with a D99N substitution was incubated with ^32^P-labeled ssRNA (top), ssRNA hybridized with oligo DNA (middle), or dsRNA (bottom). Divalent cation was supplied as indicated. As controls, *E. coli* RNase H and S1 nuclease were used. RNA was detected by urea-PAGE followed by autoradiography. Positions of the substrate RNAs are indicated to the right by arrowheads. The bands marked by an asterisk migrated at the same speed as the ssRNA. **b** SMV P3-Myc is co-immunoprecipitated with Rsv4-FLAG. Proteins were detected by anti-FLAG and anti-Myc antibodies. **c** P3-Myc from SMV-L(Q1033K) is co-immunoprecipitated with Rsv4-FLAG. C(wt) indicates strain SMV-C. **d** Rsv4(D99N) associates with SMV RdRp in infected leaves. Membrane fractions of leaf homogenates (Membrane) and immunoprecipitates using anti-FLAG antibody after solubilization (FLAG-IP) were analyzed by ^32^P incorporation (RdRp activity) and western blotting (P3 and NIb). The source data of Fig. 4a-d are provided as a Source Data file

Although the canonical function of RNase H is to degrade RNA strands of DNA–RNA hybrids, no DNA–RNA hybrid is formed during positive-strand RNA virus replication^25^. We thus investigated the substrate preference of Rsv4. Purified FLAG-tagged Rsv4 protein degraded neither single-strand RNA nor a DNA–RNA hybrid, but it showed manganese-dependent dsRNase activity (Fig. 4a). Magnesium ion did not activate the dsRNase activity of Rsv4 even at 10 mM. Thus, Rsv4 is a member of the RNase H family that degrades only dsRNA. The D99N mutation abolished dsRNase activity (Fig. 4a), suggesting that this activity is essential for SMV resistance. Positive-strand RNA viruses replicate their genomes via a complementary (negative-strand) RNA, which leads to the formation of dsRNA. Thus, a plausible mechanism of action of Rsv4 is to degrade viral dsRNA formed during replication.

Positive-strand RNA viruses replicate their genomes in a membranous replication compartment that is isolated from the cytoplasm^26,27^. Viruses thereby hide their dsRNA replicative intermediates from cytoplasmic surveillance mechanisms. Thus, Rsv4 must somehow enter the site of SMV RNA replication to be able to efficiently inhibit the replication of the virus by degrading viral dsRNA. To test this possibility, we examined whether Rsv4 physically associates with the SMV replication machinery. Since SMV-L(Q1033K) has an amino acid substitution in the P3 protein^22,28–30^, we first examined the interaction of Rsv4 with P3. Currently, the function of P3 protein of potyviruses remains obscure; however, it has transmembrane regions and interacts with viral NIb polymerase^31,32^, and it is suggested to be an essential component of the viral replication complex^33^. When Myc-tagged SMV P3 protein was co-expressed with FLAG-tagged Rsv4 protein in *N. benthamiana* leaves, P3-Myc was co-immunoprecipitated with Rsv4-FLAG from detergent-solubilized membranes of the leaf extracts (Fig. 4b). The tagged Rsv4(D99N) mutant protein (D99N-FLAG) retained the ability to bind P3-Myc (Fig. 4b), indicating that binding ability is not sufficient for resistance. We did not observe any difference between Myc-fused P3 proteins of SMV-C (wild-type) and SMV-L(Q1033K) in their ability to bind Rsv4-FLAG in the immunoprecipitation experiment (Fig. 4c). This is consistent with the above result that Rsv4 could inhibit SMV-L(Q1033K) multiplication when expressed at high levels (Fig. 2f).

Since D99N-FLAG interacted with P3 but did not inhibit SMV multiplication, we speculated that the Rsv4(D99N) mutant protein associates with the viral replication complex without affecting viral replication. We thus further examined whether a viral RdRp is co-purified with D99N-FLAG from solubilized membranes of SMV-infected leaves. Before solubilization, RdRp activity was detected in membrane fractions of D99N-FLAG–expressing and GUS (negative control)–expressing leaves inoculated with SMV, but not in those of SMV-inoculated Rsv4-FLAG–expressing or non-inoculated GUS-expressing leaves (Fig. 4d). After solubilization and immunoprecipitation with anti-FLAG antibody, RdRp activity was detected in the D99N-FLAG–immunoprecipitated fraction (Fig. 4d). SMV NIb protein (the catalytic subunit of RdRp) and P3 protein were detected in the D99N-FLAG-immunoprecipitated fraction. Thus, D99N-FLAG physically associates with a replication-catalyzing viral protein complex. These results strongly suggest that in Rsv4-expressing cells, Rsv4 enters membrane-surrounded viral replication compartments through its interaction with replication machinery; in the replication compartments, it degrades viral dsRNA replication intermediates. The results also suggest that an active potyviral RdRp contains P3 protein in addition to the NIb protein.

### Rsv4 confers broad-spectrum resistance against potyviruses

**Fig. 5:**
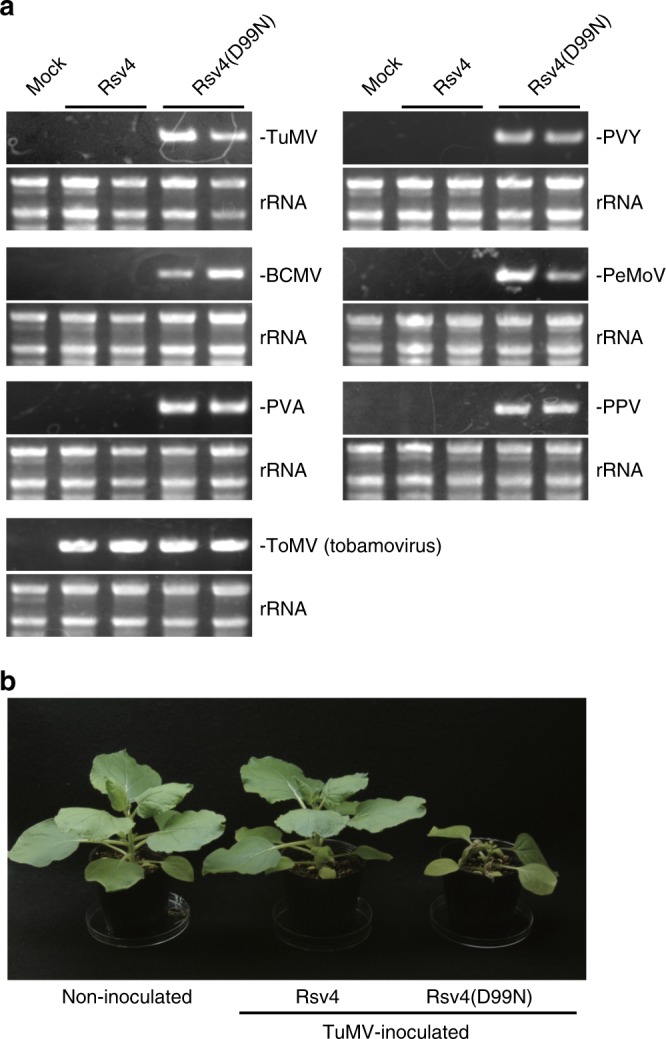
Rsv4 confers broad-spectrum resistance against potyviruses. **a** Accumulation of viral RNA in inoculated plants. Turnip mosaic virus (TuMV), potato virus Y (PVY), bean common mosaic virus (BCMV), peanut mottle virus (PeMoV), potato virus A (PVA), plum pox virus (PPV), or tomato mosaic virus (ToMV) were inoculated onto *N. benthamiana* leaves expressing Rsv4 or Rsv4(D99N), and accumulation of viral RNA in systemic leaves was detected by RT-PCR at 7–14 dpi. rRNAs were used as loading controls. **b** Symptoms of TuMV-inoculated *N. benthamiana* expressing Rsv4 or Rsv4(D99N) at 12 dpi. The source data of Fig. 5a are provided as a Source Data file

The genus *Potyvirus* consists of over 150 species, accounting for ~15% of all named plant virus species, and includes many agriculturally important viruses worldwide^34^. We tested whether Rsv4 inhibits multiplication of other potyviruses by using agroinfiltration-mediated transient expression in *N. benthamiana*. Remarkably, multiplication of TuMV, PVY, PPV, bean common mosaic virus (BCMV), potato virus A (PVA), and peanut mottle virus (PeMoV) was inhibited by Rsv4 (Fig. 5a). TuMV causes severe symptoms in *N. benthamiana* but did not induce any symptoms when inoculated onto Rsv4-expressing leaves (Fig. 5b). Thus, Rsv4 can confer broad-spectrum resistance against potyviruses. Accumulated levels of the genomic RNA of tomato mosaic virus (ToMV; genus *Tobamovirus*, family *Virgaviridae*), a positive-strand RNA virus, were comparable in the presence of Rsv4 and Rsv4(D99N) (Fig. 5a). This result disproves the possibility that Rsv4 nonspecifically degrades dsRNAs of all positive-strand RNA viruses, and provides further evidence for the importance of the interaction of Rsv4 with potyvirus replication machinery for resistance.

### Engineering dsRNases targeting viral replication sites

**Fig. 6:**
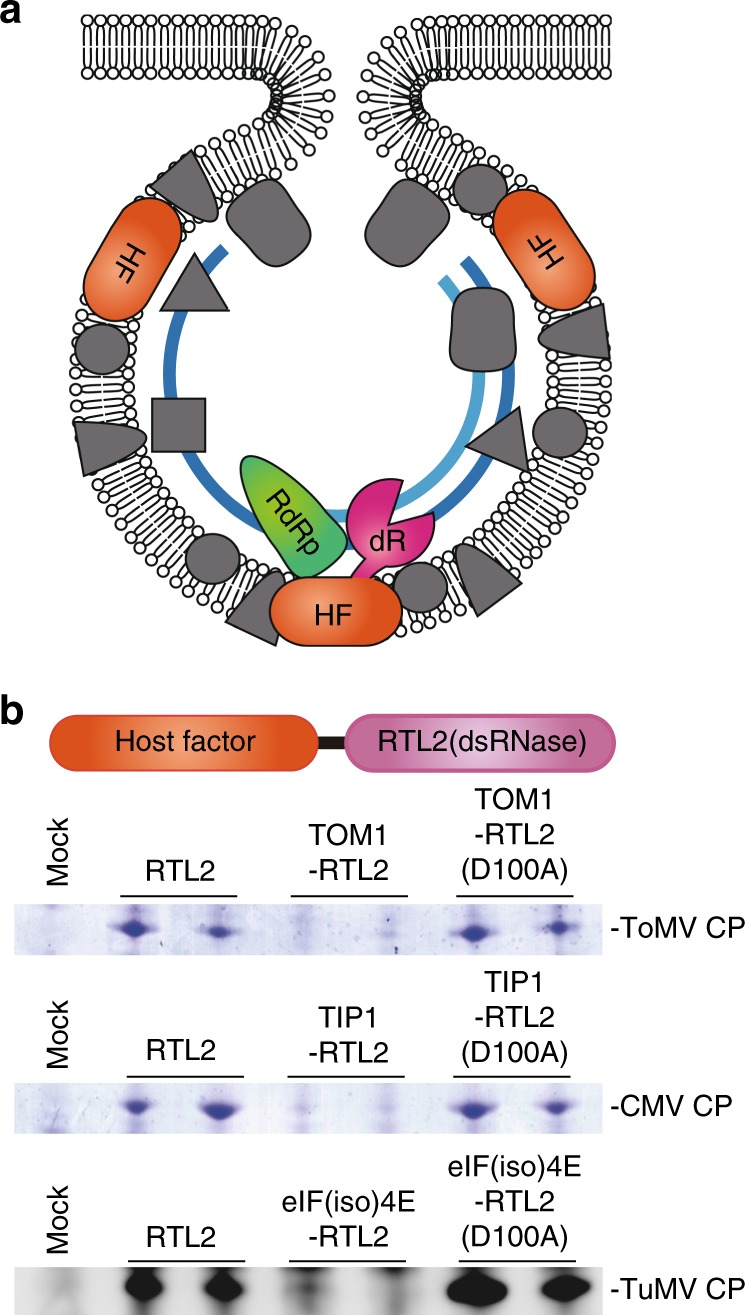
Targeted inhibition of virus multiplication by dsRNases fused with host factors involved in viral RNA replication. **a** Working model for targeted inhibition. By fusing a dsRNase (dR) with host factors (HF) included in the viral replication complex, dsRNase is delivered into the replication compartment. **b** Inhibition of multiplication of three different viruses by dsRNases designed to target the replication complexes of each virus. Accumulation of coat proteins (CP) in inoculated leaves was examined by Coomassie staining (ToMV and CMV) or western blotting (TuMV) at 2–4 dpi. The RTL2 (D100A) constructs contain a catalytic mutation in RTL2. The source data of Fig. 6b are provided as a Source Data file

Virtually all eukaryotic positive-strand RNA viruses replicate in membrane-bound replication complexes, and the dsRNA replication intermediates are formed only within the membrane compartments. The model for Rsv4 action we describe here suggests that dsRNase can confer resistance against any positive-strand RNA virus if the enzyme is delivered into the replication compartment (Fig. 6a). To examine this antiviral activity, we fused a dsRNase protein, RTL2^35^ from *Arabidopsis thaliana* (chosen because RTL2 does not have a transmembrane region), with three host proteins, each of which has been proposed to be associated with the replication complex of a specific virus: TOM1 for ToMV^36^, TIP1 for cucumber mosaic virus (CMV)^37^, and eIF(iso)4E for TuMV^38^ (genus *Tobamovirus*, *Cucumovirus*, and *Potyvirus*, respectively). When transiently expressed in *N. benthamiana* leaves, these fusion proteins greatly suppressed the multiplication of the corresponding viruses (Fig. 6b). Neither RTL2 itself nor fusion proteins with a catalytic mutation in RTL2 (D100A) inhibited multiplication of the viruses. These results reinforce the validity of the model for the virus resistance mechanism used by Rsv4, i.e., penetration of dsRNases into viral replication compartments, and suggest its applicability to designing resistance genes against viruses of interest.

## Discussion

In the present study, we identified the broad-spectrum SMV resistance gene *Rsv4* of soybean by positional cloning and demonstrated its function by transgenic expression in SMV-susceptible soybean, mutagenesis in SMV-resistant soybean, transient expression in *N. benthamiana*, and biochemical analyses. Genomic rearrangements likely caused the 3.6-kbp insertion/deletion in the *Rsv4* locus, but it is unclear exactly how the locus evolved. The geographical distribution of the 3.6-kbp insertion/deletion in soybean and *Glycine soja* shown in Fig. 3 suggests that genetic variation of this chromosome region was reduced during expansion of soybean outside its probable areas of domestication (China and Korea). Tandemly repeated SMV-susceptible *Rsv4* alleles with the 3.6-kb region also encode RNase H proteins with the DEDD(N) motif (Supplementary Fig. 5), and NM_001249088 (present in susceptible cultivars containing the 3.6-kbp insertion) inhibited SMV multiplication when overexpressed in *N. benthamiana* (Supplementary Fig. 6a). Thus, it is possible that the SMV-susceptible *Rsv4* alleles confer resistance to viruses that are not adapted to soybean, as in the case of *Tm-1/tm-1* of tomato: a susceptible allele of ToMV resistance gene *Tm-1* inhibits multiplication of viruses that are not adapted to tomato^39^.

Most dominant virus resistance genes in plants encode NB-LRR proteins that elicit a defense reaction upon recognition of the corresponding viruses. Several others encode a variety of proteins functionally divided into two groups: inhibitors of viral propagation that target RNA replication^40–42^ or systemic transport^43–45^, and modulators of plant immunity that include an enzyme sulfonating salicylic acid^46^ and RNA-dependent RNA polymerases^47,48^. We found that *Rsv4* encodes a dsRNase that associates with the SMV replication machinery. Although sequestering dsRNA is a universal feature of eukaryotic positive-strand RNA viruses and functions as a countermeasure to host antiviral immunity, Rsv4 is able to contact P3 and/or other viral proteins during replication complex assembly and enter the viral replication compartment, where it degrades viral dsRNA formed during replication.

Rsv4 interacts, whether directly or indirectly, with the SMV P3 protein (Fig. 4b). A key amino acid residue of SMV P3 that is recognized by Rsv4, Q1033, is predicted to reside within the transmembrane region^22^. Thus, Rsv4 and P3 may interact via transmembrane helix–helix interactions. It is possible that even in the presence of Rsv4, SMV makes Rsv4-free replication complexes at a low frequency, enabling viral genomic RNA to be continuously produced. The second-generation genomes could also form functional replication complexes at a low frequency. When progeny SMV genomes move to neighboring uninfected cells, they would again form Rsv4-free replication complexes at a low frequency. We propose that these processes could explain the slow SMV spread observed even in *Rsv4* plants^18^. SMV mutants whose P3 proteins have lower affinity to Rsv4 would form Rsv4-free replication complexes more frequently than wild-type strains. SMV-L(Q1033K) only partially breaks *Rsv4*-mediated resistance (Fig. 2f), and its P3 protein still had a detectable affinity to Rsv4 in the immunoprecipitation assay (Fig. 4c). Considering that SMV mutants that show virulence on *Rsv4* plants had reduced fitness in susceptible hosts^49^, P3 mutations that abolish affinity to Rsv4 may be deleterious to SMV. Future studies for quantitative measurements of the affinity between Rsv4 and P3, and their derivatives, would provide further support for our model.

As a complement to traditional breeding, biotechnological engineering has been an alternative approach to producing virus-resistant crops. Abel et al. showed that transgenic tobacco plants expressing the coat protein (CP) gene of tobacco mosaic virus are resistant to the virus^50^. This method, referred to as pathogen-derived resistance, also includes RNA interference against viral genome sequences^51^. Pathogen-derived resistance has been used to produce many virus-resistant crops^52^; however, only a handful of these transgenic plants having viral genes have been commercialized because of problems including a lack of regulatory framework in developing countries^53^. Another way to engineer virus-resistant crops is knockout/knockdown of host genes that are essential for virus multiplication^54^. This strategy does not need transgenic techniques when the knockout/knockdown is achieved using mutagenesis or genome editing, but few examples are available since loss of function of host factor genes most often affects plant growth.

We could successfully imitate Rsv4 to design custom-made antiviral proteins by fusion of a dsRNase with host proteins used by viruses for their replication. In contrast to the genetically modified virus-resistant crops created by introduction of viral genes, dsRNase-mediated resistance only requires a fusion of endogenous host genes. Thus, using this method in combination with a knock-in technology, it may be possible to create transgene-free virus-resistant crops. The resultant virus-resistant plants would grow normally if the original functions of the host factors are unaffected by fusion with dsRNase. Although transient expression of the dsRNase–host factor fusion proteins did not completely inhibit multiplication of the target viruses (Fig. 6b), knock-in plants would be expected to show higher resistance because the endogenous dsRNase-free host factors would no longer be available for the viruses. An additional advantage of this method is that plants resistant to multiple viruses may be produced by fusing dsRNase to factors commonly used by diverse viruses.

## Methods

### Plant materials

A genetic mapping population for *Rsv4* was developed from a cross between SMV-C–resistant cultivar ‘Peking’ (*Rsv4*) and SMV-C–susceptible cultivar ‘Enrei’ (*rsv4*). Both soybeans were obtained from Nagano Vegetable and Ornamental Crops Experiment Station, Shiojiri, Japan, and registered as accession numbers GmWMC084 (Peking) and GmJMC025 (Enrei) in the NIAS soybean mini core collection of the Genetic Resources Center, NARO, Tsukuba, Japan. An F_2_ mapping population consisting of 190 plants was initially used to delimit the *Rsv4* locus by using simple sequence repeat (SSR) markers. For fine mapping, five backcross populations consisting of 9320 individuals in total were further developed from F_1_ plants between ‘Enrei’ and ‘Peking’ using ‘Enrei’ as a female recurrent parent in the cross to obtain materials having recombination around *Rsv4* (Supplementary Fig. 1). Seeds with recombination detected by marker analysis were selectively grown to obtain progenies in the field or vinyl greenhouse at the Institute of Crop Sciences, Tsukuba, Japan (36°2′N, 140°8′E), from June to November. The progenies of the recombinants were used to examine resistance to SMV.

A total of 1383 cultivated (*Glycine max* [L.] Merr.) and 261 wild (*G. soja* Sieb. & Zucc.) soybean accessions from the Genetic Resources Center, NARO, in Japan and the United States Department of Agriculture (USDA) were used for diversity analyses (Supplementary Data 1). The detailed genetic relationship of the materials was reported by Kaga et al.^55^. Twenty-nine additional accessions known to have *Rsv4*^18,56^ or derived from collection with a name ‘Peking’ were obtained from USDA.

### Viruses

The following viruses were obtained from the Genetic Resources Center, NARO: SMV-A (MAFF 104063), SMV-C (104065), SMV-D (104066), SMV-E (104067), TuMV (715062), PVY (307024), BCMV (715049), PVA (307028), and PeMoV (307044). PPV was a generous gift from Drs. S. Namba, Y. Yamaji, and K. Maejima (University of Tokyo). Full-length SMV-L(Q1033K) cDNA was synthesized according to the deposited nucleotide sequence (EU871724) for construction of the infectious clone.

### Fine mapping of *Rsv4*

Publicly available SSR markers (Supplementary Data 2) were initially used to construct a linkage map around *Rsv4* using the F_2_ population as described above. Based on these results, 25 SSR markers were designed to SSR core motifs around *Rsv4* by using Primer3 with default parameters^57^ based on the reference sequence of soybean chromosomes (cultivar Williams 82, version Gmax_189^58^) obtained from the Phytozome FTP site (https://phytozome.jgi.doe.gov/pz/portal.html). To ensure specificity, primer sequences were searched against Gmax_189 to examine the number of potential binding sites, amplicon size, and location by using Genome Tester^59^ with default parameters. Total genomic DNA was extracted from young fresh leaves (0.3 g) or seed powder (50 mg) by using guanidine hydrochloride and proteinase K according to Khosla et al.^60^ with several modifications. Primers fluorescently labeled with three kinds of dye (6-FAM, HEX, and NED) were used to analyze SSR markers. Multiplex PCR consisting of several markers with different dyes was performed using a QIAGEN Multiplex PCR Kit (QIAGEN). PCR mixtures (5 µl) contained 0.2 µl of template DNA (50 ng per μl), 2.5 µl of 2 × Multiplex PCR Mix, 1 µl of 5 × Q solution, and 0.1 µl of 10 µM each primer. Touchdown PCR was programmed on a GeneAmp 9700 (Applied Biosystems) as follows: 1 cycle of initial denaturation at 95 °C for 15 min; 3 cycles of 94 °C for 30 s and 68 °C for 3 min; 3 cycles of 94 °C for 30 s and 66 °C for 3 min; 3 cycles of 94 °C for 30 s and 64 °C for 3 min; 3 cycles of 94 °C for 30 s, 62 °C for 3 min, and 1 min at 72 °C; 3 cycles of 94 °C for 30 s, 60 °C for 3 min, and 1 min at 72 °C; 3 cycles of 94 °C for 30 s, 58 °C for 3 min, and 1 min at 72 °C; 40 cycles of 30 s at 94 °C, 3 min at 55 °C, and 1 min at 72 °C; and final extension for 10 min at 72 °C. The PCR product was diluted 1/10 with water, and 1 μl was denatured in 10 µl of Hi-Di formamide with 0.2 µl of GeneScan 400HD ROX size standard and separated on an ABI 3730 capillary sequencer (Applied Biosystems). SSR marker genotypes were determined by using ABI GeneMapper ver.4.0 software (Applied Biosystems). The linkage map of *Rsv4* was constructed in JoinMap ver.4.0^61^ and the marker order of the F_2_ linkage map was determined using the maximum-likelihood mapping algorithm. The recombination frequency was converted into genetic distance (cM) using the Haldane mapping function. In addition, the linkage between the genotypes of two SSR markers, Rsv4_446 and Rsv4_320 (Supplementary Data 2), and the SMV-C resistance phenotype was examined among 68 F_2_ progenies between Tosan155 (derived from a cross between Fukusennari and Enrei) and Enrei to test whether the SMV-C resistance of Fukusennari maps to the *Rsv4* region.

For fine mapping, the genotypes of markers Rsv4_446 and Rsv4_320 were screened to obtain recombinants in the *Rsv4* region in the backcross populations. Single-nucleotide polymorphisms (SNPs) were genotyped for the recombinants by the direct-sequencing method. Primer pairs were designed around the SNPs to amplify 600–1000-bp fragments based on the procedure described above. These primer pairs are listed as Rsv4-s04-07_1, Rsv4-s035-6_71, Rsv4-86105snp_42, Rsv4-RC-1_93, and Rsv4-RC-3_55 in Supplementary Data 2. Reaction mixtures consisted of 5 μl of 2 × GoTaq® Colorless Master Mix (Promega, Madison, WI, USA), 0.2 μl of 10 μM each forward and reverse primers, and 0.2 μl of the template DNA in a total volume of 10 μl. The PCR was performed as follows: 1 cycle of initial denaturation at 95 °C for 2 min; 40 cycles of denaturation for 15 s at 95 °C, annealing for 30 s at 53 °C, and extension for 1.5 min at 60 °C. The PCR products were cleaned with ExoSAP-IT (USB Corporation, Cleveland, OH, USA). Sequencing was performed with an ABI Prism BigDye Terminator v 3.1 cycle sequencing kit (Applied Biosystems) and 5 pmol of one of the primers used to amplify the PCR product on an ABI 3730xl automated DNA analyzer (Applied Biosystems) according to the manufacturer’s manual. The sequence chromatograms were aligned with the reference sequence to determine genotypes with Sequencher 5.2 (Gene Codes Corporation, Ann Arbor, MI, USA).

Two sequence-tagged site (STS) markers, detected by the Rsv4-Seq03 and Rsv4-Seq04 primer pairs (Supplementary Data 2), were designed to amplify 6-kbp and 6.3-kbp regions, respectively, near *Rsv4*. PCR mixtures (10 µl) contained 0.2 µl of template DNA (50 ng per μl), 2 µl of 5 × PrimeSTAR GXL Buffer (Takara Bio, Shiga, Japan), 1.0 µl of PrimeSTAR GXL DNA Polymerase (1.25 U per μl), 0.8 µl of 2.5 mM dNTPs, and 0.1 µl of 20 µM each forward and reverse primers. PCR was performed on a GeneAmp PCR System 9700 (Applied Biosystems) with the following program: initial denaturation for 5 s at 98 °C; 35 cycles of denaturation for 10 s at 98 °C, annealing and extension for 30 s at 68 °C; and final extension for 30 s at 68 °C. Genotypes for the two STS markers were visually classified based on fragment size by 1% agarose gel electrophoresis: 2.5 kbp (Peking type) and 6 kbp (Enrei type) for Rsv4-Seq03; 10 kbp (Peking type) and 6.3 kbp (Enrei type) for Rsv4-Seq04.

### Virus inoculation and detection

SMV-C and SMV-L(Q1033K) cDNAs were cloned into pBI121 for agroinfection into *N. benthamiana* leaves. For inoculation of soybean plants, viruses were propagated in soybean cultivar ‘Tsuruno Tamago1’ (SMV-C, D, and E) or ‘Jack’ (SMV-A, C, and E). Each inoculum was prepared from 1 g of infected leaf tissue, which was homogenized in 10 ml of 0.1 M sodium phosphate buffer, pH 7.0, by using a mortar and pestle. Inoculation was performed before the trifoliate leaves emerged. Unifoliate soybean leaves were dusted with carborundum before inoculation, then rubbed softly with a cotton puff to distribute the inoculum, and finally rinsed with tap water. Inoculated plants were grown in a greenhouse at 18 to 25 °C for 2 to 3 weeks and classified into three phenotype classes based on symptoms of 15 to 30 plants per line: resistant (all plants were symptomless), susceptible (more than 80% of plants had mosaic symptoms), and segregating (about 25% of plants had mosaic symptoms). Additional plants were inoculated to confirm phenotype classifications when necessary. Inoculation of potyviruses other than SMV into *N. benthamiana* leaves was performed mechanically using homogenates of infected leaves as described above. Anti-SMV-CP rabbit antiserum was raised against an *E. coli*-expressed SMV-C CP. For quantification of SMV accumulation, the SMV CP bands of Western blotting were measured by ImageJ^62^. Anti-SMV-P3 and -NIb rabbit antisera were raised against synthetic peptides CFPVAVSMTGQSEDVSAQ and CVGAQYKGKKQDYFSGMD, respectively. Anti-TuMV-CP antiserum was purchased from the Japan Plant Protection Association (Tokyo, Japan). All antisera used for Western blotting were diluted at 1:1000 in TBST containing 5% (w/v) skim milk. Primers used for detection of viral RNA by RT-PCR are listed in Supplementary Data 2.

### Sequencing analysis of the *Rsv4* region

Long-range PCR was conducted to amplify a 16-kbp genomic fragment including the delimited 9.8-kbp fragment of the *Rsv4* region from ‘Peking’ using primer pair rsv4L15_L_4957 (Supplementary Data 2). PCR mixtures (10 µl) contained 0.2 µl of template DNA (50 ng per μl), 2 µl of 5 × PrimeSTAR GXL Buffer (Takara Bio), 0.2 µl of PrimeSTAR GXL DNA Polymerase (1.25 U per μl), 0.8 µl of 2.5 mM dNTPs, and 0.1 µl of 20 µM each forward and reverse primers. PCR was performed on a GeneAmp PCR System 9700 (Applied Biosystems) with the following program: initial denaturation for 5 s at 98 °C; 30 cycles of denaturation for 10 s at 98 °C, annealing and extension for 7 min 50 s at 68 °C; and final extension for 30 s at 68 °C. The sequence of the 16-kbp fragment was determined by the construction of random transposon insertion clones according to the protocol supplied with the EZ-Tn5™ < KAN-2 > Insertion Kit (Epicentre). Sequencing was performed with an ABI Prism BigDye Terminator v 3.1 cycle sequencing kit (Applied Biosystems) and 5 pmol of the sequencing primers provided with the EZ-Tn5™ kit on an ABI-3730xl automated DNA analyzer (Applied Biosystems) according to the manufacturer’s manual. The bidirectional sequence chromatograms generated from the 470 clones were assembled into a 16,159-bp contig using Sequencher 5.2.

### RNA analysis

Total RNA was isolated from young trifoliate leaves by using an RNeasy® Plant Mini Kit following the on-column DNase protocol (QIAGEN). RT-PCR was conducted by using a PrimeScript One Step RT-PCR Kit (Takara Bio) and the primer pair ORF2 (Supplementary Data 2), designed to the presumed *Rsv4* ORFs. PCR was performed on a GeneAmp PCR System 9700 (Applied Biosystems) with the following program: initial denaturation for 5 s at 98 °C; 30 cycles of denaturation for 10 s at 98 °C, annealing and extension for 7 min 50 s at 68 °C; and final extension for 30 s at 68 °C. mRNA was purified from total RNA using a MagExtractor™ kit (TOYOBO, Japan). Full-length cDNA sequence was obtained by using a GeneRacer™ Kit (Invitrogen) and nested primer sets for 5' amplification (rsv4-5-1 and rsv4-5-2) or 3' amplification (rsv4-3-1 and rsv4-3-2; Supplementary Data 2) according to the manufacturer’s manual.

### Transformation of soybean plants with *Rsv4*

The *Rsv4* RT-PCR product amplified by primer pair ORF2 was re-amplified using an adaptor primer pair, OEatg-1 and OEtga-1 (Supplementary Data 2). The fragment was double-digested by restriction enzymes *Spe*I and *Xho*I and cloned into the *Sac*I–*Xba*I sites of the pMDC123-GFP vector^63^. Then, a fragment containing the cauliflower mosaic virus (CaMV) 35 S promoter, *Rsv4* cDNA, and the NOS terminator of *Agrobacterium tumefaciens* was amplified using the primer pair P35SF1spe and TNOSR1xho (Supplementary Data 2), and the DNA fragment was double-digested by *Spe*I and *Xho*I and cloned into the *Spe*I–*Xho*I sites of the pUHR SK plasmid vector.

The genomic DNA construct for transformation was obtained as follows. PCR mixtures (50 µl) contained 2 µl of template DNA (50 ng per μl), 10 µl of 5 × PrimeSTAR HS Buffer (Takara Bio), 1 µl of PrimeSTAR HS DNA Polymerase (2.5 U per μl), 4 µl of 2.5 mM dNTPs, and 2.5 µl of 20 µM Rsv4-Seq04 primer pair (Supplementary Data 2). PCR was programmed as follows: initial denaturation for 2 s at 98 °C; 30 cycles of denaturation for 10 s at 98 °C, annealing and extension for 5 min at 68 °C; and final extension for 10 s at 68 °C. The DNA fragment was double-digested by restriction enzymes *Spe*I and *Hin*dIII and cloned into the *Spe*I–*Hin*dIII sites of the pUHR SK plasmid vector. The sequences of several clones were confirmed in advance of transformation.

The construct was introduced by biolistic transformation into cultured cells derived from immature embryos of the SMV-susceptible cultivar ‘Jack’ as described by Nishizawa et al.^64^. T0 plants derived from hygromycin-resistant and red-fluorescent embryogenic cells were grown at 28 °C day/23 °C night in a temperature-controlled glasshouse. Genomic DNA from regenerated T0 plants and T1 plants was used to confirm transformation by the constructs. Two primer pairs, pDsRed2_135 and hpt (Supplementary Data 2), were used to amplify the *DsRed* and *HPT* genes, respectively. In addition, RT-PCR for total RNA from young leaves was conducted to confirm expression of *Rsv4* in each T1 and T2 plant by using the primer pair ORF2 (Supplementary Data 2).

Long-range PCR was performed to identify germplasm with a 3.6-kbp deletion (as found in the Peking genome) using the primer pair Rsv4-Seq03 as described above. Based on the fragment sizes, germplasm was classified for the absence (PCR product size is 2.5 kbp) or presence (6 kbp) of the 3.6-kbp fragment by 1% agarose gel electrophoresis.

### Isolation of Peking *Rsv4* mutants

A mutant library was developed for Peking, and *Rsv4* mutants were identified as in Tsuda et al.^65^. Briefly, seeds of Peking were treated with the chemical mutagen EMS, and M2 seeds produced by M1 plants were treated with EMS once again to increase the mutation density. The resultant mutant library, which consisted of DNA and seeds from 1536 M2 plants, was screened by using indexed amplicon sequencing to retrieve *Rsv4* mutants. The primer pair Gm02-Rsv4-1n-5000_F and Gm02-Rsv4-1n-5001_R (Supplementary Data 2) was used to amplify a 3.6-kbp region covering *Rsv4* using the long-range PCR method described above and used for indexed amplicon sequencing. Only base changes leading to stop codon or amino acid substitutions observed in more than 2% of the aligned amplicon reads on the 3.6-kbp Peking *Rsv4* sequence were selected to isolate mutants from the library by direct sequencing using the primer pair ORF2 (Supplementary Data 2). After identification of mutants, genotypes of their progenies were confirmed by the same procedure.

### Transient expression in *N. benthamiana*

*Rsv4* cDNA was cloned into pMLH7133^66^. The D99N mutant was created by changing the 99^th^ codon, for aspartic acid (GAT), to that for asparagine (AAT). Rsv4-FLAG and D99N-FLAG were created by inserting 5'-GGAGGTGGAGATTATAAGGATGATGATGATAAG-3' before the stop codon of the respective *Rsv4* gene. P3-Myc was created by inserting the SMV-C cDNA sequence encoding the Gly766 to Gln1112 residues of the polyprotein between the initiation codon (ATG) and the Myc-tag coding sequence (5'-GGAAGATCTGAGCAGAAGCTTATTTCTGAGGAGGATCTTTGAGCTC-3') and cloned into pMLH7133. A synthetic p19 gene of tomato bushy stunt virus was cloned into pRI101-AN (TaKaRa Bio). Unless otherwise noted, a mixture of *Agrobacterium* strains that express the proteins of interest and p19 was infiltrated into *N. benthamiana* leaves using a needleless syringe at OD_600_ = 0.5 each. 1 day after infiltration, *Agrobacterium* harboring SMV cDNA was infiltrated at OD_600_ = 0.1 for SMV inoculation.

### RNase assay

*N. benthamiana* leaves expressing Rsv4-FLAG or D99N-FLAG were homogenized in an ice-cold buffer (30 mM HEPES-KOH pH 7.4, 100 mM KCl, 2 mM EDTA, 2 mM DTT, 1 tablet per 50 ml cOmplete EDTA-free Protease Inhibitor [Roche]) by using a grinder. After clarification by centrifugation (800 × *g*), the membrane fraction was pelleted by centrifugation at 16,000 × *g* for 15 min. The pellet was suspended in homogenization buffer containing 1% Triton X-100, and the solubilized proteins were purified using anti-FLAG antibody-conjugated agarose (SIGMA). FLAG-tagged proteins were eluted with 0.1 mg per ml 3× FLAG peptide in 30 mM HEPES-KOH pH 7.4, 100 mM KCl, 2 mM DTT, 0.2% Triton X-100, 3 μg per ml BSA, 1 tablet per 50 ml cOmplete EDTA-free Protease Inhibitor (Roche). The eluate was mixed with an equal volume of glycerol and stored at −30 °C. For substrate RNA production, pGEM-SMV-7610-7960 was constructed by inserting a SMV-C cDNA fragment amplified by PCR using primers SMV7610F and SMV7960R (Supplementary Data 2) into pGEM-7Zf( + ) between the *Cla*I and *Bam*HI sites. Substrate RNA was transcribed from *Bam*HI-linearized pGEM-SMV-7610-7960 using T7 RNA polymerase in the presence of [α-^32^P]CTP. A synthetic DNA (5'-GGTTCTCTAACATTTCTCTTCCAACCCACCAGTCTTCCATGAAAA-3') or RNA transcribed from *Cla*I-linearized pGEM-SMV-7610-7960 using SP6 RNA polymerase was annealed to the substrate. Digestion was performed in 30 mM HEPES-KOH pH 7.4, 50 mM KCl, 2 mM DTT, 0.1% Triton X-100 at 25 °C for 30 min. MgCl_2_ or MnCl_2_ was supplemented as indicated (Fig. 4a). *E. coli* RNase H and S1 nuclease were purchased from TaKaRa Bio, and digestion was performed in 30 mM HEPES-KOH pH 7.4, 50 mM KCl, 4 mM MgCl_2_, 2 mM DTT, 0.1% Triton X-100 for RNase H or in the supplied buffer for S1 nuclease. After the reactions, RNA was purified and analyzed by 7 M urea–10% PAGE followed by autoradiography.

### Interaction analyses of Rsv4 and SMV P3

Rsv4-FLAG and P3-Myc were co-expressed in *N. benthamiana* leaves by agroinfiltration. 3 days after infiltration, the leaves were homogenized in TR buffer (30 mM HEPES-KOH pH 7.4, 80 mM KOAc, 1.8 mM Mg(OAc)_2_, 2 mM DTT, 1 tablet per 50 ml cOmplete EDTA-free Protease Inhibitor [Roche]). After clarification by centrifugation (800×*g*), membranes were pelleted by 16,000×*g* for 15 min. The pellet was suspended in TR buffer containing 1% Triton X-100, incubated with anti-FLAG antibody at 4 °C for 2 h, washed four times with wash buffer (30 mM HEPES-KOH pH 7.4, 100 mM NaCl, 2 mM DTT, 0.2% Triton X-100, 1 tablet per 50 ml cOmplete EDTA-free Protease Inhibitor [Roche]), and eluted with wash buffer containing 0.1 mg per ml 3× FLAG peptide. The eluted samples were analyzed by western blotting using anti-Myc (Cat#16286-1-AP: Cosmo Bio, Tokyo, Japan) and anti-DYKDDDDK (Cat#018-22381: Wako Pure Chemical, Osaka, Japan) antibodies diluted at 1:1000 in TBST containing 5% skim milk.

### Viral RdRp assay

Membrane fractions of SMV-inoculated leaf extracts (6 days post-inoculation) and FLAG-purified fractions after solubilization that were prepared as described above were incubated with a template RNA (20 ng per μl in vitro-transcribed SMV full-length genomic RNA) and 5× RdRp reaction solution (5 mM ATP, 5 mM GTP, 5 mM UTP, 62.5 μM CTP, 25 mM Mg(OAc)_2_, 50 mM DTT, 0.5 mg per ml actinomycin D, 4 U per μl RNasin Plus [Promega], 1 mg per ml creatine kinase, 100 mM creatine phosphate, 370 MBq per ml [α-^32^P]CTP) at 25 °C for 90 min. Following phenol extraction and ethanol precipitation, single-stranded RNA was digested with S1 nuclease (TaKaRa Bio), and the remaining dsRNA was separated by 8 M urea–2.4% PAGE and detected by autoradiography.

### Construction of RTL2-fusion proteins

*RTL2*, *TIP1*, and *eIF(iso4E)* cDNAs were amplified from *Arabidopsis thaliana* Col-0 RNA and *TOM1* cDNA was amplified from *Nicotiana tabacum* cv. Samsun RNA using the primers listed in Supplementary Data 2. cDNAs for target proteins and RTL2 were fused by overlap PCR with ten glycine codons (GGT×10) as a linker and cloned into pMLH7133. As negative controls, a mutation causing D100A (GAT to GCT) of RTL2 was introduced into each fusion protein by site-directed mutagenesis, and RTL2 alone was cloned into pMLH7133. A mixture of *Agrobacterium* strains that express p19 and one of the fusion proteins (OD_600_ = 0.2 and 0.5, respectively) was infiltrated into *N. benthamiana* leaves using a needleless syringe. Two days after infiltration, ToMV, CMV, or TuMV was mechanically inoculated.

### Diversity analysis

Multiple alignments of nucleotide and protein sequences were constructed using a progressive sequence alignment tool^67^ implemented in the CLC Genomics Workbench with default parameter values. A BLAST (blastp) search to find sequences similar to Rsv4 was performed against the nonredundant (nr) protein database maintained by NCBI with an *E*-value cutoff of 3e^−10^. A phylogenetic tree was constructed by neighbor joining with 1000 bootstrap replications based on ‘Kimura 80’ and ‘Kimura protein’^68^ genetic distances for nucleotide and protein sequences, respectively. Genome assemblies of azuki bean (*Vigna angularis* [Willd.] Ohwi and Ohashi), Vangularis_v1; pigeon pea (*Cajanus cajan* [L] Millsp.), ICPL87119_v1; and common bean (*Phaseolus vulgaris* L.), Pvulgaris_218_v1.0, were obtained from the *Vigna* Genome Server (VigGS; https://viggs.dna.affrc.go.jp), Legume Information System (LIS; https://legumeinfo.org/organism/Cajanus/cajan), and Phytozome FTP site, respectively. Dot plot analysis^69^ implemented in the CLC Genomics Workbench was used to find similar regions on the same chromosome, among orthologous soybean chromosomes, and between genomes of different species using default parameters and a window size of 9.

### Reporting summary

Further information on research design is available in the Nature Research Reporting Summary linked to this article.

## Supplementary information

Supplementary Information

Reporting Summary

Description of Additional Supplementary Files

Supplementary Data 1

Supplementary Data 2

Source Data

## Data Availability

Data supporting the findings of this work are available within the paper and its Supplementary Information files. A reporting summary for this Article is available as a Supplementary Information file. The datasets generated and analyzed during the current study are available from the corresponding author upon request. The nucleotide sequences of SMV-C cDNA and Peking *Rsv4* mRNA are deposited in DDBJ under accession numbers LC323107 and LC325439, respectively. The genomic DNA sequences for the *Rsv4* regions of different soybean germplasms are deposited under accession numbers DDBJ LC325364 –LC325438 and GenBank accession numbers MN178650-MN178656. The source data underlying Figs. 2b–f, 4a–d, 5a, and 6b and Supplementary Figs. 3c, 6a, and 6b are provided as a Source Data file.

## References

[1] DeWitte-Orr SJ, Mossman KL (2010). dsRNA and the innate antiviral immune response. Future Virol..

[2] Pumplin N, Voinnet O (2013). RNA silencing suppression by plant pathogens: defence, counter-defence and counter-counter-defence. Nat. Rev. Micro..

[3] De Ronde, D., Butterbach, P. & Kormelink, R. Dominant resistance against plant viruses. *Front Plant Sci***. 5,** 307 (2014). 10.3389/fpls.2014.00307 PMC4073217 25018765

[4] Revers, F. & García, J. A. In *Advances in Virus Research* Vol. 92 (eds Maramorosch, K. & Mettenleiter, T. C.) 101–199 (Academic Press, 2015).

[5] Solomon-Blackburn RM, Barker H (2001). Breeding virus resistant potatoes (*Solanum tuberosum*): a review of traditional and molecular approaches. Heredity.

[6] Kyle MM, Palloix A (1997). Proposed revision of nomenclature for potyvirusresistance genes in Capsicum. Euphytica.

[7] Sharma, S. K. & Tripathi, S. In *Plant Virus–Host Interaction* (eds Gaur, R. K., Hohn, T. & Sharma, P.) 177–194 (Academic Press, Boston, 2014).

[8] Hartmann W, Neumüller M (2006). Breeding for resistance: breeding for Plum pox virus resistant plums (*Prunus domestica* L.) in Germany. EPPO Bull..

[9] Li G., Lv H., Zhang S., Zhang S., Li F., Zhang H., Qian W., Fang Z., Sun R. (2019). TuMV management for brassica crops through host resistance: retrospect and prospects. Plant Pathology.

[10] Jain, S., McPhee, K., Kumar, A., Rouf Mir, R. & Singh, R. In *Agricultural Sustainability* (eds Bhullar, G. S. & Bhullar, N. K.) 221–244 (Academic Press, San Diego, 2013).

[11] Ross JP (1977). Effect of aphid-transmitted soybean mosaic virus on yields of closely related resistant and susceptible soybean lines. Crop Sci..

[12] Hajimorad MR, Domier LL, Tolin SA, Whitham SA, Saghai Maroof MA (2018). *Soybean mosaic virus*: a successful potyvirus with a wide distribution but restricted natural host range. Mol. Plant Pathol..

[13] Hajimorad MR, Hill JH (2001). *Rsv1*-mediated resistance against *Soybean mosaic virus*-N is hypersensitive response-independent at inoculation site, but has the potential to initiate a hypersensitive response-like mechanism. Mol. Plant-Microbe Interact..

[14] Suh SJ (2011). The *Rsv3* locus conferring resistance to *Soybean mosaic virus* is associated with a cluster of coiled-coil nucleotide-binding leucine-rich repeat genes. Plant Gen..

[15] Cho E-K, Goodman RM (1979). Strains of soybean mosaic virus: classification based on virulence in resistant soybean cultivars. Phytopathology.

[16] Klepadlo M (2017). Two tightly linked genes for soybean mosaic virus resistance in soybean. Crop Sci..

[17] Tran P-T, Widyasari K, Seo J-K, Kim K-H (2018). Isolation and validation of a candidate *Rsv3* gene from a soybean genotype that confers strain-specific resistance to soybean mosaic virus. Virology.

[18] Gunduz I, Buss GR, Chen P, Tolin SA (2004). Genetic and phenotypic analysis of *Soybean mosaic virus* resistance in PI 88788 soybean. Phytopathology.

[19] Saghai Maroof MA (2010). Fine mapping and candidate gene discovery of the soybean mosaic virus resistance gene, *Rsv4*. Plant Gen..

[20] Wang D (2011). Fine mapping and analyses of *R*_*SC8*_ resistance candidate genes to soybean mosaic virus in soybean. Theor. Appl. Genet..

[21] Ilut DC (2016). Identification of haplotypes at the *Rsv4* genomic region in soybean associated with durable resistance to soybean mosaic virus. Theor. Appl. Genet..

[22] Chowda-Reddy RV (2011). Mutations in the P3 protein of *Soybean mosaic virus* G2 isolates determine virulence on *Rsv4*-genotype soybean. Mol. Plant-Microbe Interact..

[23] Shakiba E (2013). Inheritance and allelic relationships of resistance genes for *Soybean mosaic virus* in ‘Corsica’ and ‘Beeson’ soybean. Crop Sci..

[24] Nowotny M, Gaidamakov SA, Crouch RJ, Yang W (2005). Crystal structures of RNase H bound to an RNA/DNA hybrid: substrate specificity and metal-dependent catalysis. Cell.

[25] Buck, K. W. In *Advances in Virus Research* (eds Maramorosch, K., Murphy, F. A. & Shatkin, A. J.) 159–251 (Academic Press, 1996).

[26] den Boon JA, Ahlquist P (2010). Organelle-like membrane compartmentalization of positive-strand RNA virus replication factories. Annu. Rev. Microbiol..

[27] Paul D, Bartenschlager R (2013). Architecture and biogenesis of plus-strand RNA virus replication factories. World J. Virol..

[28] Ahangaran A, Habibi MK, Mohammadi G-HM, Winter S, García-Arenal F (2013). Analysis of *Soybean mosaic virus* genetic diversity in Iran allows the characterization of a new mutation resulting in overcoming *Rsv4*-resistance. J. Gen. Virol..

[29] Khatabi B, Fajolu OL, Wen RH, Hajimorad MR (2012). Evaluation of North American isolates of *Soybean mosaic virus* for gain of virulence on *Rsv*-genotype soybeans with special emphasis on resistance-breaking determinants on *Rsv4*. Mol. Plant Pathol..

[30] Wang Y, Khatabi B, Hajimorad MR (2015). Amino acid substitution in P3 of *Soybean mosaic virus* to convert avirulence to virulence on *Rsv4*-genotype soybean is influenced by the genetic composition of P3. Mol. Plant Pathol..

[31] Merits A, Guo D, Järvekülg L, Saarma M (1999). Biochemical and genetic evidence for interactions between potato A potyvirus-encoded proteins P1 and P3 and proteins of the putative replication complex. Virology.

[32] Guo D, Rajamäki M-L, Saarma M, Valkonen JPT (2001). Towards a protein interaction map of potyviruses: protein interaction matrixes of two potyviruses based on the yeast two-hybrid system. J. Gen. Virol..

[33] Cui X (2017). The C-terminal region of the *Turnip mosaic virus* P3 protein is essential for viral infection via targeting P3 to the viral replication complex. Virology.

[34] Wylie SJ (2017). ICTV virus taxonomy profile: *Potyviridae*. J. Gen. Virol..

[35] Kiyota E (2011). An Arabidopsis RNase III-like protein, AtRTL2, cleaves double-stranded RNA in vitro. J. Plant Res..

[36] Yamanaka T (2000). *TOM1*, an *Arabidopsis* gene required for efficient multiplication of a tobamovirus, encodes a putative transmembrane protein. Proc. Natl Acad. Sci..

[37] Kim MJ, Kim HR, Paek K-H (2006). Arabidopsis tonoplast proteins TIP1 and TIP2 interact with the cucumber mosaic virus 1a replication protein. J. Gen. Virol..

[38] Beauchemin C, Boutet N, Laliberté J-F (2007). Visualization of the interaction between the precursors of VPg, the viral protein linked to the genome of *Turnip mosaic virus*, and the translation eukaryotic initiation factor iso 4E in planta. J. Virol..

[39] Ishibashi K, Naito S, Meshi T, Ishikawa M (2009). An inhibitory interaction between viral and cellular proteins underlies the resistance of tomato to nonadapted tobamoviruses. Proc. Natl Acad. Sci..

[40] Ishibashi K, Masuda K, Naito S, Meshi T, Ishikawa M (2007). An inhibitor of viral RNA replication is encoded by a plant resistance gene. Proc. Natl Acad. Sci..

[41] Yoshida, T. et al. The plant non-canonical antiviral resistance protein JAX1 inhibits potexviral replication by targeting the viral RNA-dependent RNA polymerase. *J. Virol.* **93**, e01506–e01518 (2018). 10.1128/JVI.01506-18 PMC6340027 30429349

[42] Liu Q (2017). An atypical thioredoxin imparts early resistance to *Sugarcane mosaic virus* in maize. Mol. Plant.

[43] Chisholm ST, Parra MA, Anderberg RJ, Carrington JC (2001). Arabidopsis *RTM1* and *RTM2* genes function in phloem to restrict long-distance movement of tobacco etch virus. Plant Physiol..

[44] Whitham SA, Anderberg RJ, Chisholm ST, Carrington JC (2000). Arabidopsis *RTM2* gene is necessary for specific restriction of tobacco etch virus and encodes an unusual small heat shock-like protein. Plant Cell.

[45] Cosson P (2010). *RTM3*, which controls long-distance movement of potyviruses, is a member of a new plant gene family encoding a meprin and TRAF homology domain-containing protein. Plant Physiol..

[46] Wang Q (2014). *STV11* encodes a sulphotransferase and confers durable resistance to rice stripe virus. Nat. Commun..

[47] Verlaan MG (2013). The tomato yellow leaf curl virus resistance genes *Ty-1* and *Ty-3* are allelic and code for DFDGD-class RNA–dependent RNA polymerases. PLoS Genet..

[48] Butterbach P (2014). Tomato yellow leaf curl virus resistance by *Ty-1* involves increased cytosine methylation of viral genomes and is compromised by cucumber mosaic virus infection. Proc. Natl Acad. Sci..

[49] Wang Y, Hajimorad MR (2016). Gain of virulence by *Soybean mosaic virus* on *Rsv4*-genotype soybeans is associated with a relative fitness loss in a susceptible host. Mol. Plant Pathol..

[50] Abel P (1986). Delay of disease development in transgenic plants that express the tobacco mosaic virus coat protein gene. Science.

[51] Pooggin MM (2017). RNAi-mediated resistance to viruses: a critical assessment of methodologies. Curr. Opin. Virol..

[52] Lindbo JA, Falk BW (2017). The impact of “coat protein-mediated virus resistance” in applied plant pathology and basic research. Phytopathology.

[53] Kreuze JF, Valkonen JPT (2017). Utilization of engineered resistance to viruses in crops of the developing world, with emphasis on sub-Saharan Africa. Curr. Opin. Virol..

[54] Gal-On A, Fuchs M, Gray S (2017). Generation of novel resistance genes using mutation and targeted gene editing. Curr. Opin. Virol..

[55] Kaga A (2012). Evaluation of soybean germplasm conserved in NIAS genebank and development of mini core collections. Breed. Sci..

[56] Shi A, Chen P, Zheng C, Hou A, Zhang B (2008). A PCR-based marker for the *Rsv1* locus conferring resistance to *Soybean mosaic virus*. Crop Sci..

[57] Rozen, S. & Skaletsky, H. In *Bioinformatics**Methods and Protocols* (eds Misener, S. & Krawetz, S. A.) 365–386 (Humana Press, Totowa, 1999).

[58] Schmutz, J. et al. Genome sequence of the paleopolyploid soybean. *Nature* **463**, 178–183 (2010). 10.1038/nature08670 20075913

[59] Andreson R, Reppo E, Kaplinski L, Remm M (2006). GENOMEMASKER package for designing unique genomic PCR primers. BMC Bioinforma..

[60] Khosla S, Augustus M, Brahmachari V (1999). Sex-specific organisation of middle repetitive DNA sequences in the mealybug Planococcus lilacinus. Nucleic Acids Res..

[61] Ooijen, J. W. *JoinMap 4.0: software for the calculation of genetic linkage maps in experimental populations* (Kyazma BV, Wageningen, Netherlands, 2006).

[62] Schneider CA, Rasband WS, Eliceiri KW (2012). NIH Image to ImageJ: 25 years of image analysis. Nat. Methods.

[63] Liu B (2010). The soybean stem growth habit gene *Dt1* is an ortholog of arabidopsis *TERMINAL FLOWER1*. Plant Physiol..

[64] Nishizawa K, Kita Y, Kitayama M, Ishimoto M (2006). A red fluorescent protein, DsRed2, as a visual reporter for transient expression and stable transformation in soybean. Plant Cell Rep..

[65] Tsuda M (2015). Construction of a high-density mutant library in soybean and development of a mutant retrieval method using amplicon sequencing. BMC Genom..

[66] Mitsuhara I (1996). Efficient promoter cassettes for enhanced expression of foregin genes in dicotyledonous and monocotyledonous plants. Plant Cell Physiol..

[67] Feng D-F, Doolittle RF (1987). Progressive sequence alignment as a prerequisitetto correct phylogenetic trees. J. Mol. Evol..

[68] Kimura M (1980). A simple method for estimating evolutionary rates of base substitutions through comparative studies of nucleotide sequences. J. Mol. Evol..

[69] Maizel JV, Lenk RP (1981). Enhanced graphic matrix analysis of nucleic acid and protein sequences. Proc. Natl Acad. Sci. USA.

