# Supplementary material for: Soybean antiviral immunity conferred by dsRNase targets the viral replication complex
Source: Nat Commun. 2019 Sep 27;10:4033. doi: 10.1038/s41467-019-12052-5 (PMC6764979; doi:10.1038/s41467-019-12052-5)
Supplement: Supplementary file 1 — Supplementary Information [file 41467_2019_12052_MOESM1_ESM.pdf]

Soybean antiviral immunity conferred by dsRNase targets the viral replication complex

Ishibashi *et al*.

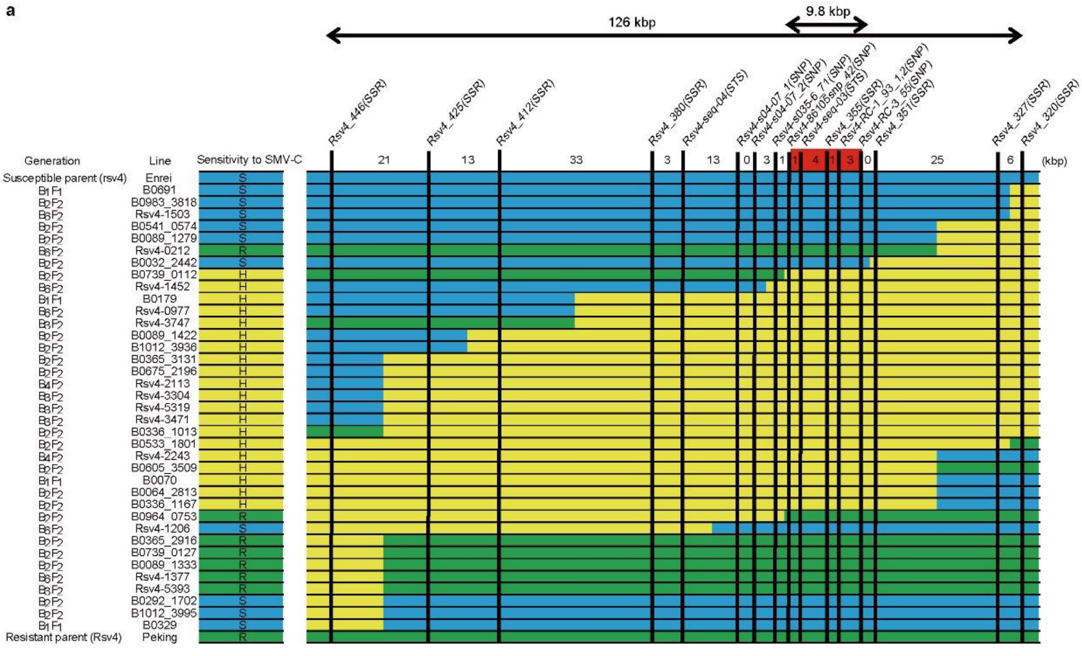

b

Genotyped generation	Number of plants genotyped	Number of recombinants	SMV-tested generation
BC_1F_1	1,046	4	BC_1F_2
BC_2F_2	3,840	20	BC_2F_3
BC_3F_2	2,514	5	BC_3F_3
BC_4F_2	384	2	BC_4F_3
BC_6F_2	1,536	6	BC_6F_3
Total	9,320	37	

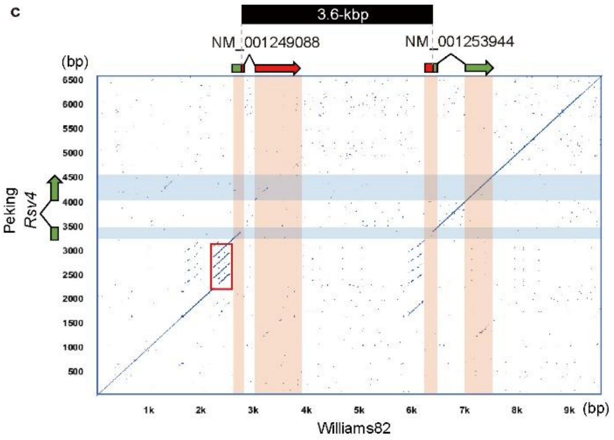

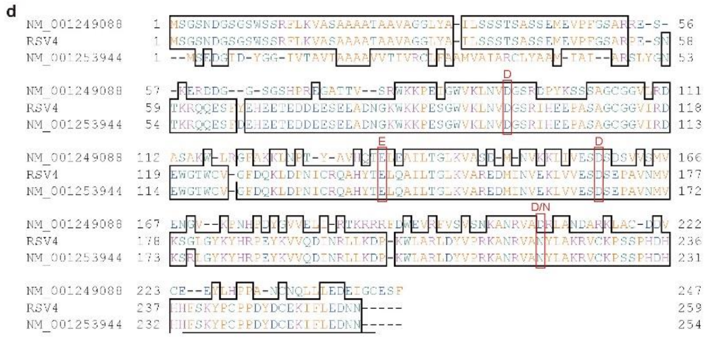

Supplementary Fig. 1. Graphical genotype of the *Rsv4* region. (a) Recombination breakpoints around the *Rsv4* region on soybean chromosome 2 are indicated by black vertical lines corresponding to 16 molecular markers (indicated above the chromosome diagrams). Genotypes: blue, Enrei; green, Peking; yellow, heterozygous. Sensitivity of the recombinants to SMV infection, shown immediately to the left of the chromosome maps, was evaluated using their progenies and matched perfectly with the genotype in the 9.8-kbp region indicated in red. (b) Summary of the materials used for fine mapping. (c) Dot plot comparing the *Rsv4* genomic region of Peking with that of Williams 82. Dark blue dots and lines indicate genomic sequences that have more than 88% sequence identity between the two genomes. Green arrows or arrow parts indicate the *Rsv4* ORF (left) and the Williams 82 ORF regions (top) having high similarity with the *Rsv4* ORF. Red arrow parts indicate Williams 82 ORF regions having low similarity with the *Rsv4* ORF. The 5′ upstream sequence of NM_001249088 in Williams 82 was

repeatedly found in the Peking genome (red box). The black box at the top indicates the location of the 3.6-kbp insertion in Williams 82 relative to Peking. (d) Alignment of the predicted amino acid sequences of Rsv4, NM_001249088, and NM_001253944. DEDD(N) motif is indicated by red boxes.

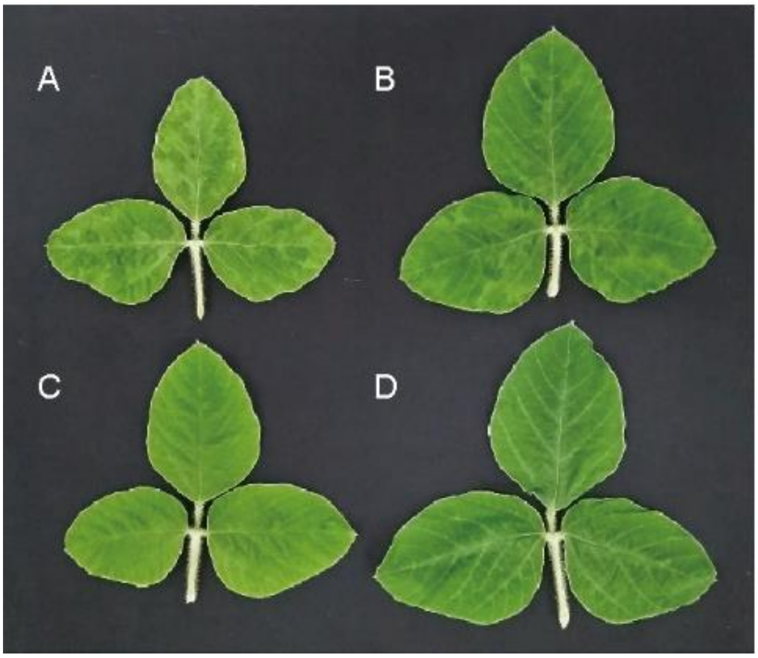

Supplementary Fig. 2. Symptoms of the fourth leaves of SMV-C-inoculated Peking mutant plants at 15 dpi. A, PeM-1355 (Pro49Ser); B, PeM-0220 (Pro49Leu); C, Peking (wild type [Pro49]); D, PeM-0220 (segregant with a wild-type sequence [Pro49Pro]).

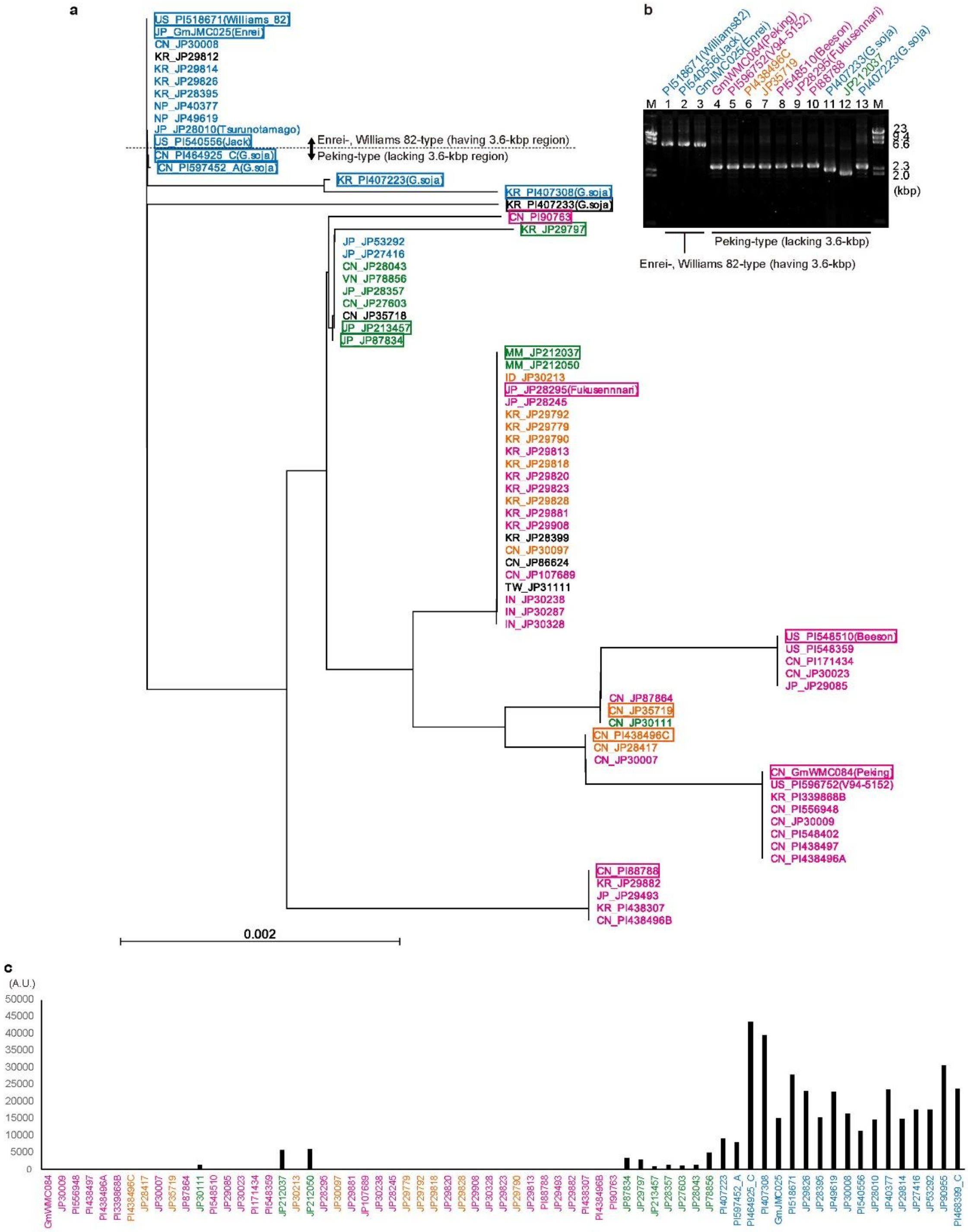

Supplementary Fig. 3. Genetic variation in *Rsv4* among soybean germplasm. Colors of accession names indicate SMV susceptibility: cyan, SMV CP was highly accumulated at 13 dpi; green, SMV CP was detectable at 13 dpi, orange, SMV CP was not detectable at 13 dpi but detectable at 21 dpi, magenta, SMV CP was not detectable until 21 dpi, and black, not examined. (a) Relationships between amino acid sequence variations in Rsv4 and SMV sensitivity are illustrated on a phylogenetic tree. The Rsv4 amino acid sequences of 63 accessions containing the 3.6-kbp deletion were aligned with those of the corresponding region (which is split into two ORFs by the 3.6-kbp insertion) in 11 accessions. Accession names in boxes indicate representative accessions. Each accession name in the tree starts with a country code followed by the accession number. The amino acid sequence of one wild soybean (*G. soja*) accession, PI464925_C, had the same sequence as Williams 82 and Enrei except for the absence of the region corresponding to the 3.6-kbp deletion. (b) Amplification of the fragment encompassing the *Rsv4* ORF from representative accessions by PCR using an STS marker, Rsv4-Seq03. A 6-kbp PCR

fragment was amplified from several SMV-susceptible genetic resources containing the 3.6-kbp insertion (lanes 1–3). Among the accessions lacking the 3.6-kb insertion, a shorter (2.4-kbp) fragment was amplified from genetic resources that strongly inhibited SMV-C multiplication (lanes 4–10), whereas fragments from accessions that allowed SMV-CP accumulation at 13 dpi (lanes 11–13) varied in size. M: Lambda DNA *Hin*dIII digest. (c) Quantification of SMV CP accumulation at 13 dpi. Equal amounts of leaf patches from systemic leaves of three plants for each accession were mixed and homogenized for Western blotting. SMV CP bands were detected by LuminoGraph III (ATTO, Tokyo, Japan) and quantified using ImageJ. The source data of Supplementary Figure 3c are provided as a Source Data file.

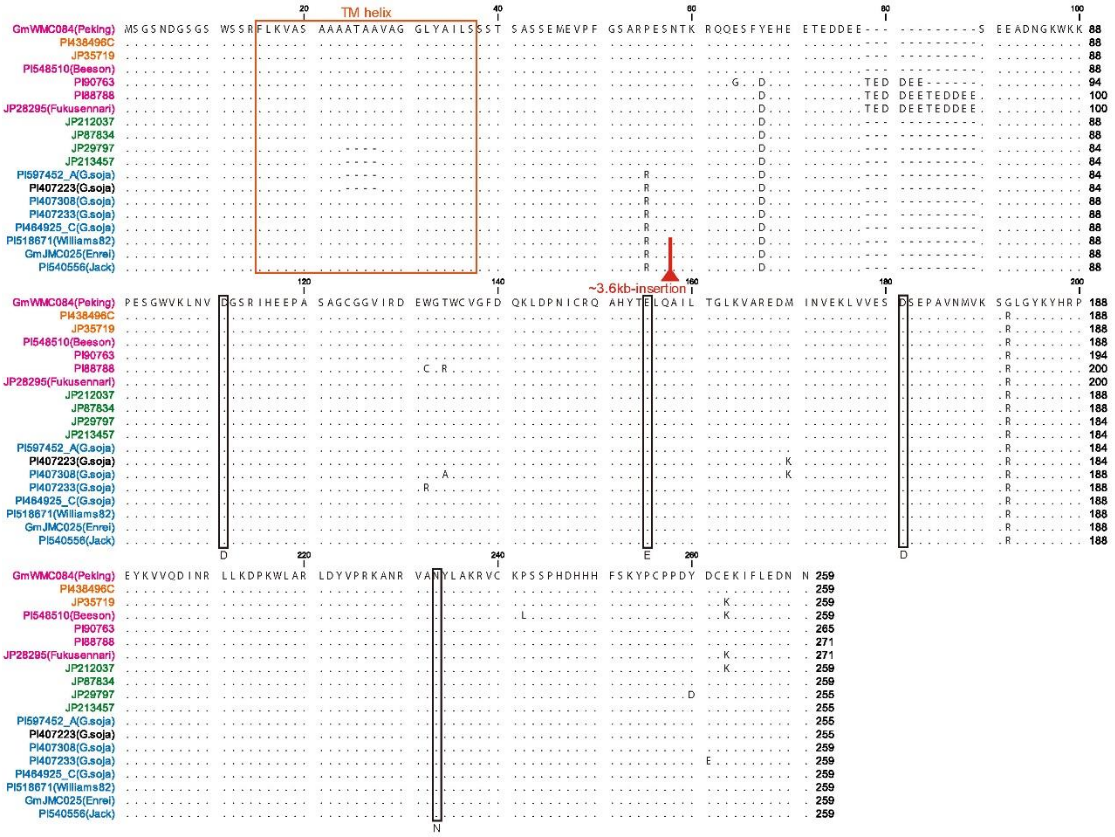

Supplementary Fig. 4. Alignment of Rsv4 amino acid sequences of the 19 representative accessions. Accession names are colored as in Supplementary Fig. 3. Deduced amino acid sequences of Rsv4 from sixteen accessions lacking the 3.6-kbp region, and the corresponding region of three accessions (bottom three rows) having the 3.6-kbp region are aligned.

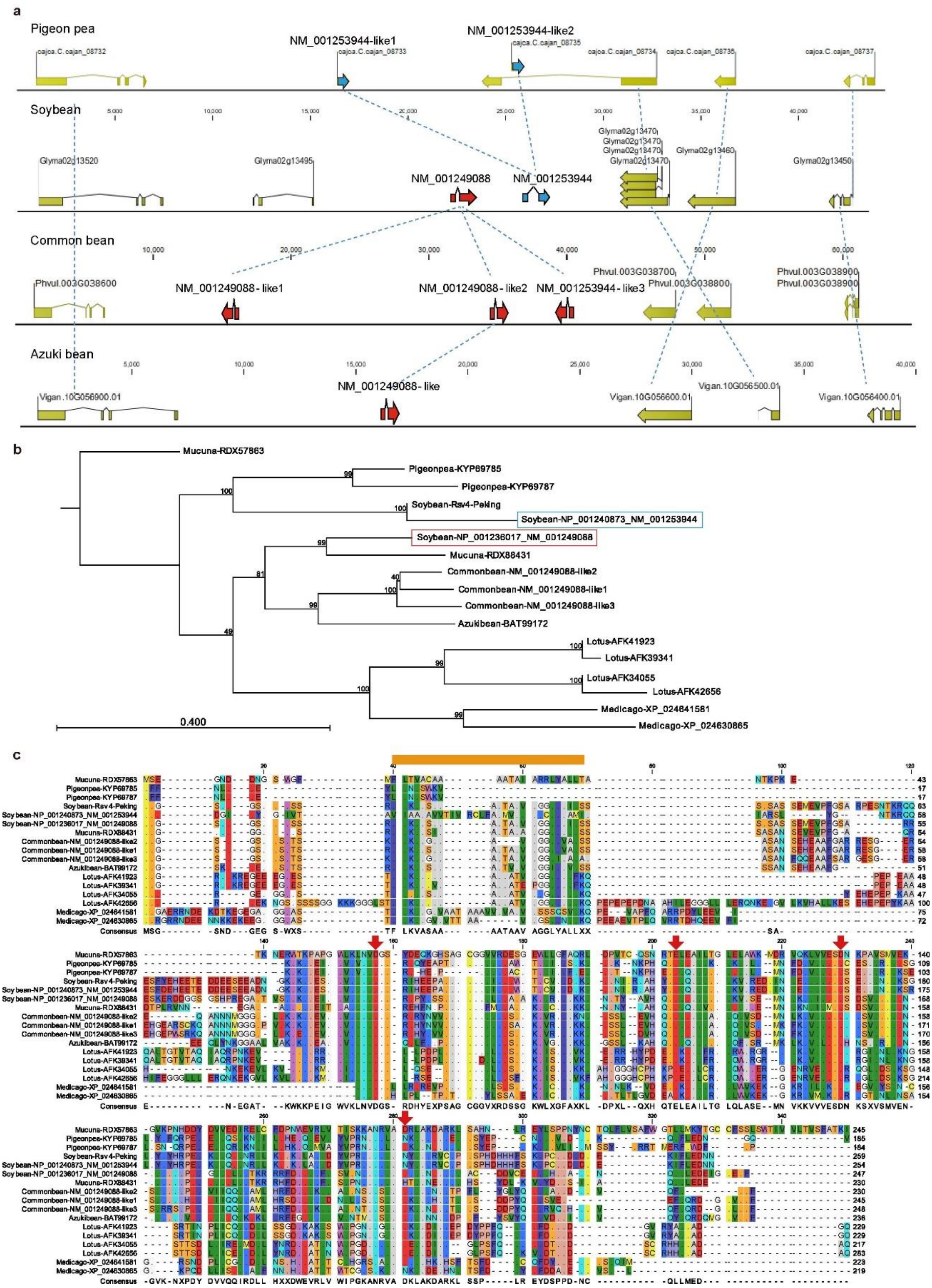

Supplementary Fig. 5. Comparison of *Rsv4* locus among legumes. (a) Comparison of *Rsv4* genomic region among four legumes. ORFs with similar nucleotide sequences are connected by dotted lines. The ORF sequence NM_001249088 of soybean (red) was identified in the azuki bean and common bean genomes, whereas *Rsv4* (not shown) and ORF NM_001253944 (blue) were identified in the pigeon pea genome. Other ORFs in the region are shown in yellow. (b) Phylogenetic relationships and (c) amino acid alignment of predicted protein sequences from *Rsv4* homologs. Sequence names in red and blue boxes in (b) indicate soybean ORFs NM_001249088 and NM_001253944, respectively. Orange bar and red arrows in (c) indicate the positions of the conserved transmembrane helix and DEDD/DEDN motif, respectively.

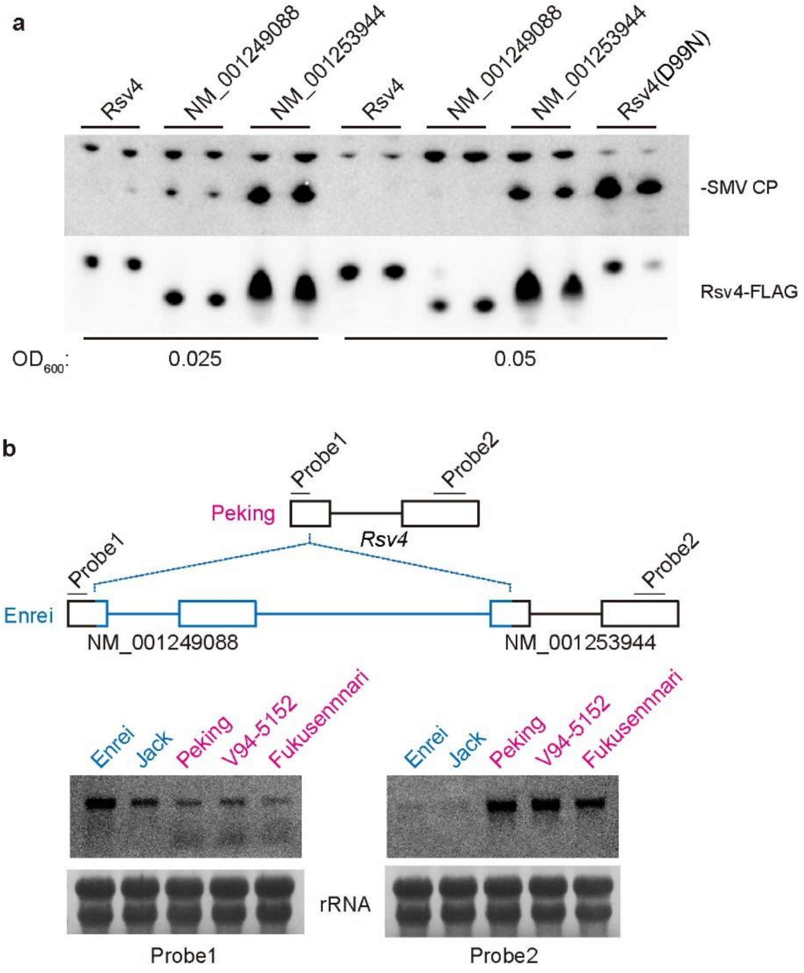

Supplementary Fig. 6. An *Rsv4* allele in SMV-susceptible soybean encodes a potential inhibitor of potyvirus multiplication. (a) Inhibition of SMV multiplication by NM_001249088. FLAG-tagged Rsv4, NM_001249088, and NM_001253944 were expressed in *N. benthamiana* by agroinfiltration at indicated concentrations. One day after infiltration, *Agrobacterium* harboring SMV cDNA was infiltrated at OD_{600} = 0.01. Western blotting using anti-SMV CP and anti-FLAG was performed at 6 dpi. Each lane represents an individual plant. (b) Detection of mRNA for Rsv4, NM_001249088, and NM_001253944 by Northern blot hybridization. Twenty micrograms of total RNA were electrophoresed, and the mRNAs were detected by digoxigenin-labeled probes hybridize to the indicated positions. Ribosomal RNAs were detected by methylene blue staining as loading controls. The source data are provided as a Source Data file.

Supplementary Table 1. Phenotypic segregation of SMV-C-inoculated Peking mutant lines with mutations in the *Rsv4* gene.

	Line name					
	PeM-1211	PeM-0484	PeM-0220	PeM-1355	PeM-1080	Peking
Position in CDS (bp)	359	359	146	145	329	NA
Genotype of wild type	G/G	G/G	C/C	C/C	C/C	NA
Genotype of the mutant	G/A	G/A	C/T	T/T	T/T	NA
Amino acid change	Trp120*	Trp120*	Pro49Leu	Pro49Ser	Ala110Val	NA
Genotype of mutation site in progeny (Number of plants)						
Wild type (W)	5	4	5	NA	NA	16
Heterozygous (H)	9	9	11	NA	NA	NA
Mutant type (M)	6	4	5	8	8	NA
Total	20	17	21	8	8	16
Chi square (1:2:1)	0.30	0.06	0.05	NA	NA	NA
p value (d.f.=2)	0.86	0.97	0.98	NA	NA	NA
Phenotype in progeny (Number of plants)						
Resistant (R)	14	13	16	0	8	16
Susceptible (S)	6	4	5	8	0	0
Total	20	17	21	8	8	16
Chi square (3:1)	0.27	0.02	0.02	NA	NA	NA
p value (d.f.=1)	0.61	0.89	0.90	NA	NA	NA
Chi square test for linkage and independence of segregation between genotype and phenotype						
R, W+H	14	13	16	NA	NA	NA
R, M	0	0	0	NA	NA	NA
S, W+H	0	0	0	NA	NA	NA
S, M	6	4	5	NA	NA	NA
Total	20	17	21	NA	NA	NA
Chi square (12:0:0:4)	0.27	0.02	0.02	NA	NA	NA
p value (d.f.=3)	0.97	1.00	1.00	NA	NA	NA
Chi square (9:3:3:1)	26.22	15.73	19.72	NA	NA	NA
p value (d.f.=3)	8.6E-06	1.3E-03	1.9E-04	NA	NA	NA

Chi-square tests for Mendelian segregation ratio and genetic linkage between susceptibility and genotype in the progeny of mutant lines were examined.

Asterisks denote nonsense mutations generating stop codons.

12:0:0:4 and 9:3:3:1 correspond to tests for complete linkage and independent segregation, respectively.
