# Supplementary material for: Soybean antiviral immunity conferred by dsRNase targets the viral replication complex
Source: Nat Commun. 2019 Sep 27;10:4033. doi: 10.1038/s41467-019-12052-5 (PMC6764979; doi:10.1038/s41467-019-12052-5)
Supplement: Supplementary file 3 — Description of Additional Supplementary Files [file 41467_2019_12052_MOESM3_ESM.docx]

**Description of Additional Supplementary Files**

File Name: Supplementary Data 1
Description: List of cultivated and wild soybean germplasms used for diversity analysis of the *Rsv4* locus.

File Name: Supplementary Data 2

Description: Oligonucleotides used in this study.
